# Microstructural Evolution Governing the Creep Resistance of Grade 92 Steel Under Wide-Temperature Heat Treatment: From Ferrite Recovery to Dynamic Precipitation Strengthening

**DOI:** 10.3390/ma19061101

**Published:** 2026-03-12

**Authors:** Yinsheng He, Hongyu Zhou, Liming Xu, Keesam Shin

**Affiliations:** 1 National Center for Materials Service Safety, University of Science and Technology Beijing, Beijing 100083, China; hyzhou@ustb.edu.cn; 2 School of Mechanical and Energy Engineering, Ningbo Tech University, Ningbo 315100, China; xlm2023@nbt.edu.cn; 3 School Materials Science and Engineering, Changwon National University, Changwon 51140, Republic of Korea; keesamgg@gmail.com

**Keywords:** Grade 92 steel, thermal excursion, creep resistance, dynamic precipitation, hardness-creep life decoupling, PWHT

## Abstract

**Highlights:**

- Hardness and strength show a non-monotonic evolution across 760–1000 °C.
- Creep life hits a minimum at 850 °C and recovers to the AR level at 950 °C.
- Decoupling occurs as 1000 °C has highest hardness but 1/4 the creep life of 950 °C sample.
- Rejuvenation at 950 °C restores integrity of damaged T92 steel *via* dynamic precipitation.

**Abstract:**

Thermal excursions during post-weld heat treatment (PWHT) and on-site fabrication frequently compromise the integrity of Grade 92 steel. While hardness fluctuations are documented, the correlation between initial properties and long-term creep stability remains controversial. This study aims to evaluate the relationship between thermal history and subsequent creep performance. Heat treatments of T92 steel across a wide temperature range (760–1000 °C) were performed, followed by creep tests at 600 °C/130 MPa and microstructural characterization. Results reveal a non-monotonic evolution of hardness and strength, reaching a minimum at 850 °C due to martensitic lath recovery into ferrite, but nearly doubling the as-received (AR) values above 900 °C due to fresh martensite formation. Creep life drops to a minimum at 850 °C and recovers to the AR level at 950 °C. A significant “decoupling” occurs at 1000 °C, where the sample possesses the highest hardness but only exhibits one-fourth the life of the 950 °C sample. Superior performance stems from the retained M_23_C_6_ and its dynamic precipitation, which pins dislocations to form micro-lath structures. Conversely, 1000 °C facilitates full carbide dissolution, accelerating dislocation recovery. These findings emphasize precise PWHT control and demonstrate that a 950 °C rejuvenation treatment can restore over-tempered or damaged components.

## 1. Introduction

Grade 92 tempered martensite ferritic steel is widely utilized for critical components in ultra-supercritical (USC) power plants, such as main steam pipes and headers, due to its excellent creep resistance and thermal conductivity at temperatures up to 620 °C and 30 MPa [1,2,3]. The superior creep strength of Grade 92 steel is primarily derived from its stabilized martensitic lath structure, which is reinforced by fine M_23_C_6_ carbides and MX carbonitrides. Additionally, the precipitation of the W-rich Laves phase during service plays a dual role in modulating long-term creep response by contributing to subgrain boundary stability [1,2,3].

During the construction and maintenance of power plants, welding is an indispensable joining technology. To mitigate high residual stresses and restore the necessary fracture toughness, post-weld heat treatment (PWHT) is mandatory. International engineering codes [4,5,6], strictly mandate a PWHT temperature window of 730–770 °C for T/P92 steel [7,8]. However, localized thermal excursions frequently occur during on-site fabrication, where temperatures exceed the lower critical transformation temperature (*A_c_*_1_), approximately 800–830 °C. Field failure investigations have revealed a significant engineering paradox whereby regions subjected to such excursions often exhibit Vickers hardness exceeding 400 HV, yet suffer from premature creep failure within a fraction of the design life [9,10,11]. This fundamental decoupling between initial hardness and long-term creep life poses a significant concern for the safety assessment of power plant components, as conventional hardness-based life prediction models may become inherently unreliable.

Extensive research has been conducted to understand the microstructural response of 9Cr steels to varying thermal cycles [3,12,13]. Scholars have established that as the heating temperature increases from the sub-critical to the fully austenitized regime, the room-temperature strength and hardness follow a distinct “V-shaped” evolution pattern. Tokunaga et al. [14] and He et al. [15] demonstrated that between 800 °C and 850 °C, the drastic drop in strength is primarily governed by the recovery of martensitic laths into equiaxed ferrite and the associated loss of boundary strengthening. Conversely, heating above the *A_c_*_3_ (890–940 °C) resets the matrix into fresh martensite with a high dislocation density, causing a sharp rebound in hardness.

While the detrimental effects of excessive austenite grain growth and over-tempering on creep ductility have been documented [16], the specific role of secondary-phase kinetics remains unclear. For instance, the dynamic precipitation of M_23_C_6_ and the Laves phase (Fe_2_W) provides a supplementary pinning effect on subgrain boundaries [17]. However, the potential discrepancy between the peak hardness and intrinsic microstructural instability under creep remains to be elucidated. Moreover, the capacity of the dynamically formed Laves phase to compensate for the accelerated recovery of a fresh martensitic matrix requires further clarification, particularly in the absence of stable pre-existing carbides.

Prior studies on 9Cr steels mostly correlate microstructural evolution with mechanical properties during standard PWHT optimization. However, the quantitative impact of such thermal excursions on subsequent long-term creep resistance is rarely reported. Specifically, it remains unclear whether the higher hardness achieved through re-austenitization necessarily correlates with the restoration of creep life. This study investigates T92 steel subjected to heat treatments from 760 °C to 1000 °C, covering the sub-critical, inter-critical (*A_c_*_1_–*A_c_*_3_), and fully austenitized regimes. By comparing the tensile and hardness properties with the creep rupture life at 600 °C/130 MPa, we clarify the microstructural origin of the hardness-creep life decoupling. These findings provide a scientific basis for the life assessment and maintenance of Grade 92 components subjected to abnormal thermal excursions.

## 2. Materials and Methods

The material investigated was a commercial T92 steel tube with a nominal composition (wt.%) of 9.06Cr-1.78W-0.45Mo-0.21V-0.06Nb-0.1C-0.04N-0.003B (Fe balance). The as-received (AR) tube was in a normalized and tempered condition. To induce diverse microstructural variations, specimens were heated to temperatures ranging from 760 °C to 1000 °C, a range designed to encompass the critical transformation temperatures *A_c_*_1_ (800–830 °C) and *A_c_*_3_ (890–940 °C). The heat treatment was conducted at a constant heating rate of 200 °C/h with a holding time of two hours at the target temperature. Subsequently, the specimens were cooled at the same rate (200 °C/h) down to 400 °C, followed by air cooling to room temperature. According to the Continuous Cooling Transformation (CCT) characteristics of T/P 92 steel [18], this cooling rate is sufficient to ensure a complete martensitic transformation for samples austenitized above the *A_c_*_3_ temperature.

Vickers microhardness was measured under a 0.2 kgf load and 10 s dwell time, with ten indentations per specimen to ensure statistical reliability. Room-temperature tensile tests and creep rupture tests were performed following ASTM standards [19,20]. Round bar specimens with a gauge length of 36 mm and a diameter of 4 mm were utilized for both tests. Creep tests were conducted at 600 °C under a constant load of 130 MPa using an ATS (Applied Test Systems, Butler, PA, USA) lever-arm testing machine. The as-received condition was creep tested in duplicate to ensure a reliable baseline. Furthermore, supplementary tests at 100 MPa were conducted to verify the consistency of the identified creep life trends for selected heat-treated conditions.

Microstructural evolution was characterized using a JEOL JSM-7001F field-emission scanning electron microscope (FE-SEM, JEOL Ltd., Tokyo, Japan) and a JEM-2100F transmission electron microscope (TEM, JEOL Ltd., Tokyo, Japan). Electron backscatter diffraction (EBSD) data were acquired using a TSL system attached to an FE-SEM (MIRA II LMH, TESCAN, Brno, Czech Republic) to quantify grain boundary distributions and martensitic lath structures. Back-scattered electron (BSE) imaging was employed to differentiate W-rich Laves phase from M_23_C_6_ carbides [21]. Quantitative analysis was performed using an Image-Pro Plus 5.0 on BSE images (magnifications of 10,000× to 20,000×) from three representative regions to determine the mean diameter, number density, and area fraction of the Laves phase. TEM thin foils were prepared by twin-jet electropolishing in a solution of 5% perchloric acid and 95% acetic acid solution at −20 °C, while precipitates were further analyzed using carbon extraction replicas [22].

## 3. Results

### 3.1. Correspondence and Divergence Between Strength and Creep Rupture Life

**Figure 1:**
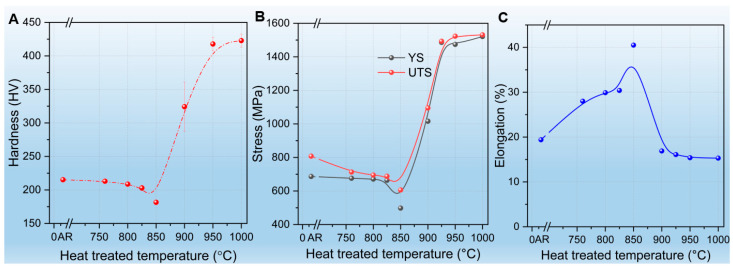
Mechanical properties of T92 steel after heat treatment at temperatures from 760 °C to 1000 °C: (**A**) Vickers microhardness; (**B**) yield strength (YS) and ultimate tensile strength (UTS); (**C**) elongation.

Figure 1A shows the variation in microhardness (Vickers) as a function of the heat treatment temperature. The hardness exhibits a non-monotonic evolution, remaining relatively stable at approximately 215 HV within the sub-critical and early inter-critical regimes (760 °C to 825 °C). A distinct softening occurs as the temperature increases to 850 °C, where the hardness reaches a minimum value of 182 HV. This softening behavior aligns with reported trends for 9Cr steels in the temperature ranges of 776–856 °C [22].

As the temperature rises beyond 900 °C into the austenitized regime (>*A_c_*_3_), a sharp hardening trend emerges. The hardness nearly doubles, reaching a plateau of approximately 420 HV at 900–1000 °C. This substantial increase indicates a complete microstructural reset to a fresh martensitic structure with a high dislocation density upon cooling [23]. While some studies suggest that higher normalizing temperatures (e.g., above 1040 °C) are required for full phase stability in Grade 91/92 steels [14], the present results demonstrate that a significant hardening effect and structural transformation are already well-established at 1000 °C.

Figure 1B presents the yield strength (YS) and ultimate tensile strength (UTS) at room temperature as a function of the heat treatment temperature. Consistent with the hardness trend, both YS and UTS reach their lowest points at 850 °C (YS = 498 MPa), a profile similar to the reported trend for T/P92 tubes subjected to similar thermal cycles [24]. A dramatic surge in strength occurs between 850 °C and 900 °C; the UTS jumps from approximately 600 MPa to over 1100 MPa, eventually exceeding 1500 MPa at 1000°C.

Figure 1C illustrates the change in elongation as a function of temperature. A remarkable ductility peak of approximately 40% is achieved at 850 °C, which precisely corresponds to the strength minimum observed in Figure 1B. This high ductility reflects the enhanced strain accommodation capacity of the recovered ferritic matrix [25]. However, this superior ductility is rapidly lost as the temperature enters the fully austenitized regime (>900 °C), where the elongation drops and stabilizes at roughly 15%. These results reveal a clear trade-off between strength and ductility, demonstrating that while the microstructural reset at high temperatures significantly boosts initial strength, it simultaneously leads to a brittle constitutive behavior compared to the optimal combinations achieved through standard processing [25,26].

**Figure 2:**
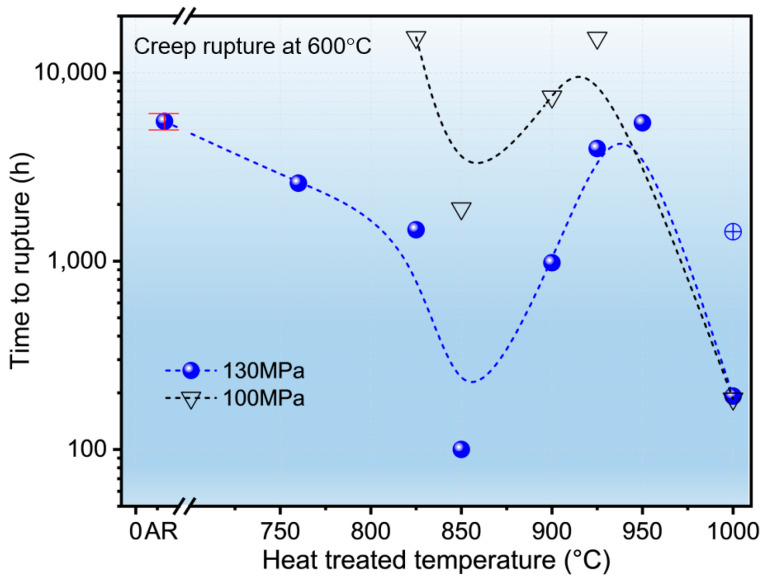
Creep rupture life of T92 steel at 600 °C under the constant loads of 130 MPa and 100 MPa following heat treatments between 760 °C and 1000 °C. Error bar for the AR condition represents the range of rupture lives from duplicate tests. Note the significant scatter for the 1000 °C samples (192 h and 1432 h under 130 MPa), where the circled plus symbol (⊕) denotes an occasional stochastic case reflecting the inherent instability of the fully re-austenitized matrix.

Figure 2 shows the creep rupture life (in log scale) of the T92 steel at 600 °C under constant loads of 130 MPa and 100 MPa as a function of the heat treatment temperature. The results exhibit a distinct and consistent non-monotonic trend where the shortest rupture life of 100 h (at 130 MPa) occurs at 850 °C, while the performance at 950 °C reaches 5422 h, which is comparable to the 5520 h recorded for the AR state. In the temperature range from 760 °C to 825 °C, a slow and steady decrease in creep life is observed, dropping from 2592 h to 1467 h. This gradual decline mirrors the degradation patterns observed during long-term thermal ageing or service exposure, where the progressive evolution of the martensitic substructure leads to a reduction in rupture strength [27,28].

A pronounced reduction in creep life is recorded as the temperature reaches 850 °C for both stress levels, consistent with the detrimental effects of microstructural instability reported in T92 steel subjected to critical thermal exposures [27,29].

As the temperature is further increased to 950 °C, a significant recovery of the creep properties is observed. The rupture life of 5422 h (at 130 MPa) indicates that this temperature effectively resets the martensitic matrix, similar to the effects of standard normalizing treatments designed to restore the baseline creep strength of 9Cr steels [26,30]. However, a further increase in the heat treatment temperature to 1000 °C results in a subsequent sharp decrease in creep resistance to 192 h (130 MPa). This level of performance is close to the minimum recorded at 850 °C, suggesting that while high-temperature treatments can significantly boost initial hardness, they may simultaneously introduce structural vulnerabilities that facilitate earlier crack incubation or accelerated creep deformation under constant load [28,30].

Importantly, the creep life evolution at 100 MPa exhibits a highly consistent non-monotonic trend with the 130 MPa results, reinforcing the conclusion that this behavior is fundamentally governed by the initial microstructural state. Notably, a significant scatter was observed for the 1000 °C specimen at 130 MPa (with one test reaching 1432 h), the stochastic nature of which will be further discussed in Section 4 in the context of microstructural instability. These findings highlight that the long-term resistance to deformation at high temperatures is highly sensitive to the initial thermal history, even when the room-temperature mechanical properties appear satisfactory [1,26,30].

### 3.2. Phase Transformation and Precipitates Dissolution Evolution Before Creep

To understand the observed variations in mechanical and creep properties, the microstructural evolution of T92 steel subjected to different heat treatments was investigated before and after creep rupture at 600 °C and 130 MPa, utilizing combined EBSD and TEM observations (including thin foil and carbon extraction replicas).

**Figure 3:**
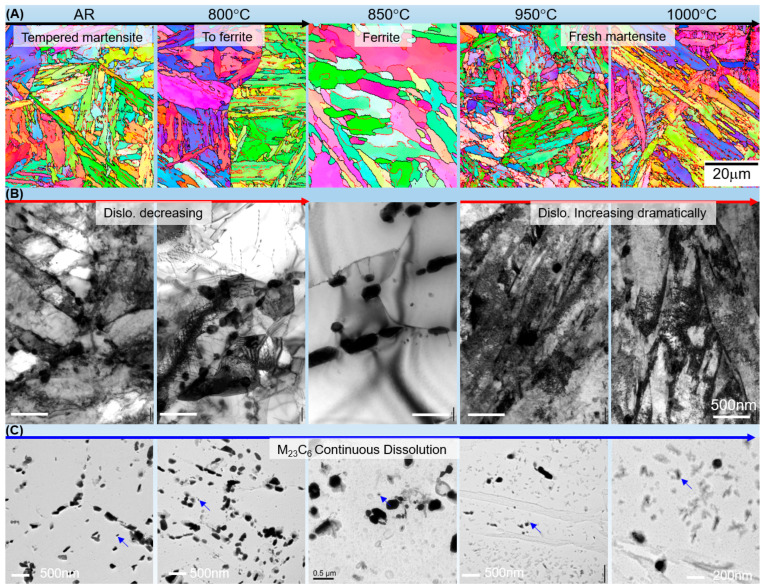
Microstructural evolution of T92 steel as a function of heat treatment temperature: (**A**) EBSD inverse pole figure (IPF) maps showing the transition from a tempered martensite lath structure to ferrite (at 850 °C) and the formation of fresh martensite (above 950 °C); (**B**) TEM thin foil images illustrating the evolution of dislocation cells, aggregation, and their interaction with M_23_C_6_ particles; (**C**) TEM images of carbon extraction replicas revealing the coarsening and subsequent dissolution of M_23_C_6_ carbides at elevated temperatures. The typical MX particles (approximately 20 to 50 nm), marked by arrows in (**C**), remains thermodynamically stable across the investigated temperature range.

Figure 3A presents the EBSD inverse pole figure (IPF) maps, illustrating the evolution of the matrix grain structure. In the AR state, the material exhibits a typical tempered martensite lath structure [1,31,32]. As the heat treatment temperature increases to 800 °C and 850 °C, a significant transition occurs where the lath morphology gradually disappears, being replaced by recovered ferrite grains. This structural transition from martensitic laths to a recovered ferritic matrix in the inter-critical range is consistent with the microstructural response typically observed in 9–12% Cr steels [15,23]. However, once the temperature exceeds the *A_c_*_3_ point (950 °C and 1000 °C), a full phase transformation back to fresh martensite is observed, which is characterized by a refined lath structure with high-angle boundaries.

The evolution of the dislocation structure (cells) and its interaction with precipitate was further revealed by TEM thin foil imaging (Figure 3B). From the AR to 850 °C, the dislocation (cells) and segregations, which appear as dark contrast, show a continuous decreasing trend. This reaches a minimum at 850 °C, where the matrix appears nearly dislocation-free within the large ferrite grains. This recovery process involves the annihilation of transformation dislocations and the migration of subgrain boundaries, leading to a significant reduction in stored energy [13,30,33]. Conversely, for samples treated at 950 °C and 1000 °C, the dislocation (cell) density increases dramatically due to the martensitic transformation during cooling. It is also worth noting that the black particles in Figure 3B, identified as M_23_C_6_ carbides, show a decreasing trend in number density with increasing temperature.

TEM observation of the carbon extraction replicas was further used to confirm the precipitate evolution (Figure 3C). In the AR state, fine M_23_C_6_ carbides are densely distributed along the prior austenite grain boundaries (PAGBs) and lath boundaries. As the temperature rises, these carbides undergo continuous coarsening and dissolution. At 850 °C, the carbides are significantly coarsened and sparsely distributed, losing their pinning effect on boundaries. Above 950 °C, the M_23_C_6_ carbides largely dissolve into the matrix during austenitization, following the thermodynamic solubility limits of chromium and carbon in austenite [1,34]. While some fine precipitates may re-form during cooling, the overall quantity of coarse carbides is significantly reduced compared to the lower-temperature treatments.

In contrast, fine MX carbonitrides remain thermodynamically stable across the investigated temperature range, as their dissolution temperatures typically exceed 1150 °C. Since the MX phase remains essentially unchanged after heat treatment (representative MX particles are marked by arrows in Figure 3C) and during subsequent creep at 600 °C, its contribution to creep behavior is considered a constant factor [1]. Therefore, the following analysis focuses on the dynamic evolution of M_23_C_6_ carbides and the Laves phase as the primary variables influencing creep performance.

**Figure 4:**
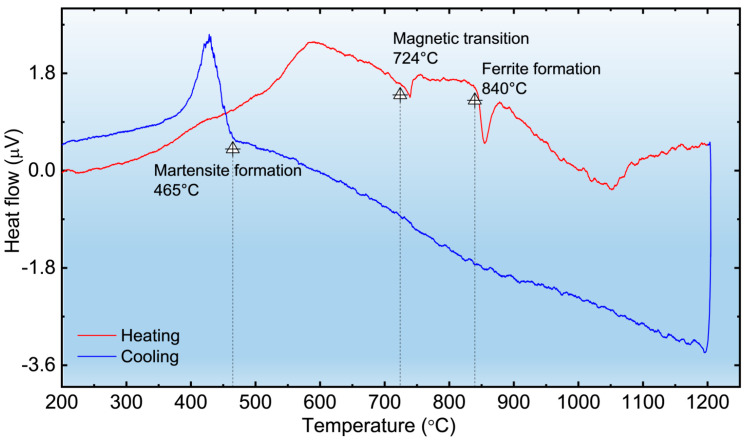
DTA curves of the AR T92 steel during heating and cooling at the same rates and holding time as the heat treatments. The characteristic temperatures for the Curie point (724 °C), the α⟶γ transformation peak (840 °C), and the martensite formation peak (465 °C) are indicated [23].

To provide a thermodynamic basis for the observed microstructural changes and mechanical properties, differential thermal analysis (DTA) was conducted on the AR sample, as shown in Figure 4. During the heating process, an endothermic signal at 724 °C corresponds to the magnetic transition (Curie temperature) [23]. A significant endothermic peak follows at approximately 840 °C, identifying the onset of the α→γ phase transformation and the intensive dissolution of carbides. Upon cooling, a sharp exothermic peak at 465 °C represents the martensite start temperature (*M_s_*) [35]. These characteristic temperatures are in excellent agreement with the microstructural transitions observed in Figure 3.

Overall, the microstructural evolution from 760 °C to 1000 °C is characterized by a “recovery-transformation” cycle of the matrix and the “coarsening-dissolution” of precipitates. The 850 °C condition represents a microstructural extreme with a coarse ferritic matrix. While higher temperatures (950–1000 °C) restore the martensitic structure and high dislocation density, the depletion of initial M_23_C_6_ carbides through dissolution could weaken the long-term creep resistance, particularly at 1000 °C as will be discussed further in Section 4.

### 3.3. Phase Transformation and Precipitates Evolution After Creep

**Figure 5:**
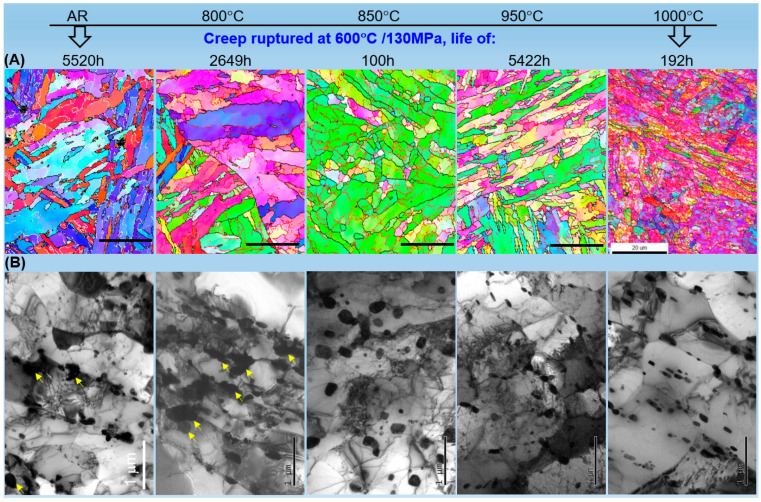
Microstructural characterization of heat-treated T92 steel after creep rupture at 600 °C and 130 MPa: (**A**) EBSD IPF maps illustrating grain evolution and fragmentation; (**B**) TEM images showing the dislocation-precipitate interactions, dynamic precipitation of M_23_C_6_ in 950–1000 °C samples, and the formation of Laves phase in long-term samples (as marked arrows in (**B**)).

The microstructural evolution after creep rupture at 600 °C and 130 MPa is presented in Figure 5, where the gauge sections were analyzed to compare with the initial states in Figure 3.

Figure 5A displays the EBSD IPF maps, revealing distinct grain and boundary evolution across different samples. In the AR-crept sample, compared to the initial tempered martensite in Figure 3A, the microstructure maintains a recognizable lath morphology, though noticeable lath widening and subgrain formation occurred after a long-term exposure of 5520 h. As the heat treatment temperature rises to 760 °C, a similar trend is observed; however, certain local regions exhibit an accelerated growth of lath structures, leading to a heterogeneous grain size distribution after 2649 h of creep.

A dramatic change in the ferrite grain structure is observed in the 850 °C-crept sample. While it initially consisted of coarse and elongated ferrite grains (Figure 3A), the microstructure after only 100 h of creep exhibits significant grain refinement and fragmentation. This phenomenon is attributed to the interaction between intense plastic deformation and pre-existing carbides. Under the current dislocation-dominated creep deformation regime, the pre-existing M_23_C_6_ carbides (particularly those within the grain interiors) serve as potent barriers that impede dislocation motion, leading to localized dislocation pile-ups. These dislocation tangles subsequently evolve into new sub-boundaries (low-angle boundaries), effectively partitioning the original large ferrite grains into smaller ones. This carbide-assisted deformation-induced grain fragmentation is a characteristic response of recovered ferritic structures under high-stress creep conditions [10,27].

As the heat treatment temperature increases into the fully austenitized region (950 °C and 1000 °C), the initial fresh martensite (Figure 3A) undergoes substantial recovery during creep. For the 950 °C–crept sample (5422 h), the lath structure persists and shows a morphology similar to that of the AR-crept sample, albeit with a slightly coarser feature. In contrast, the 1000 °C sample, despite its much shorter rupture life of 192 h, exhibits more pronounced lath widening and a severe breakdown of the martensitic structure compared to the 950 °C sample. It is critical to note that while both 950 °C and 1000 °C samples initially possessed a fresh martensitic matrix, their creep lives differ by nearly fourfold. This discrepancy is closely linked to the initial state of M_23_C_6_ precipitates, which will be further discussed in Section 4.

The microstructural degradation and secondary phase evolution were further examined using TEM thin-foil imaging (Figure 5B), providing a microscopic explanation for the grain evolution observed via EBSD and the pivotal role of carbides in governing matrix stability through their interaction with dislocations and boundaries. In the AR and 800 °C crept samples, the M_23_C_6_ carbides underwent significant coarsening relative to their initial states. Notably, as the heat treatment temperature increases toward 800 °C, the degree of M_23_C_6_ coarsening during creep appears to decrease, transitioning from a state of severe over-aging to a more stable configuration.

This trend reaches a unique extreme in the 850 °C-crept sample. Although the M_23_C_6_ carbides were already severely coarsened during the initial heat treatment (Figure 3B), they show negligible further growth after the creep test. This is primarily attributed to the extremely short rupture life (100 h), which provided insufficient time for further diffusion-controlled coarsening. Interestingly, these pre-existing M_23_C_6_ particles played a crucial role in the grain refinement of the 850 °C sample mentioned earlier. As observed in the TEM micrographs (Figure 5B), these carbides are distributed along the ferrite grain boundaries, low-angle boundaries, and even within the dislocation networks. They acted as potent obstacles that effectively pinned both the migrating boundaries and the newly formed low-angle boundaries during plastic deformation. This demonstrates a powerful Zener pinning effect [36], where the carbides stabilize the matrix and facilitate grain fragmentation even within a fully recovered ferritic matrix [17].

For the samples treated in the austenitized regime (950 °C and 1000 °C), TEM imaging reveals a transition from the initial fresh martensite to a well-defined tempered lath structure (Figure 5A). More importantly, a high density of fine M_23_C_6_ carbides is observed decorating the lath boundaries and prior austenite grain boundaries (PAGBs). The number density of these carbides is significantly higher than in their post-heat treatment state (Figure 3B), yet their size remains much smaller than those in the AR to 850 °C samples. This observation indicates a robust process of dynamic precipitation during the creep exposure. Such dynamic precipitation of M_23_C_6_ and Laves phase particles is often triggered by the high density of nucleation sites provided by the martensitic transformation induced dislocations [1,17]. This dynamic strengthening mechanism allows the 950 °C samples to maintain structural stability and achieve creep resistance comparable to the AR state. However, the lower initial carbide volume fraction at 1000 °C (due to prior dissolution) ultimately limits its long-term endurance.

Notably, in the TEM images of AR and 800 °C crept samples, micron-sized Laves phase particles (exhibiting black-contrast) are observed at the grain boundaries and triple junctions, as marked by arrows in Figure 5B. These W-rich precipitates, typically associated with long-term thermal exposure, will be further characterized in the subsequent BSE analyses to discuss their impact on solid-solution strengthening depletion [1,2].

**Figure 6:**
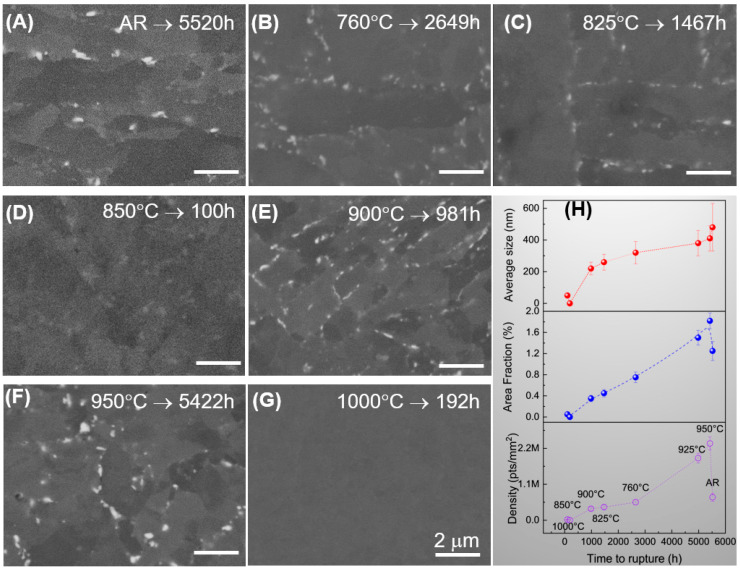
BSE images and quantitative analysis of Laves phase after creep rupture: (**A**–**G**) Distribution of Laves phase in creep ruptured samples; (**H**) Statistical results of number density, average diameter, and area fraction for the crept samples.

The precipitation of secondary phases, particularly the W-rich Laves phase, was further analyzed using BSE imaging to elucidate its role in the long-term creep resistance. As shown in Figure 6A–G, the bright-contrast particles, identified as the Laves phase due to their high atomic weight, exhibit significant variations in size and distribution depending on the initial heat treatment temperature and the resulting creep life. A dominant time-dependent growth is evident, but the precipitation kinetics are strongly modulated by the initial heat treatment temperature (Figure 6H).

In the samples treated at lower temperatures (AR to 825 °C), the Laves phase follows a typical coarsening path. For instance, the AR-5520 h crept sample exhibits the largest average size of 480 nm with an area fraction of 1.25%. In contrast, for the 850 °C-100 h and 1000 °C-192 h crept specimens, only trace amounts of Laves phase were observed (<0.05% area fraction), confirming that these premature failures occurred within the incubation period for W-rich intermetallic nucleation.

The quantitative evidence for enhanced Laves phase formation after creep is founded in the 950 °C-5422 h crept sample. Despite having a creep life nearly identical to the AR-5520 h sample, the 950 °C sample exhibits a nearly three-fold increase in number density (2.35 *vs.* 0.7 × 10^6^ pts/mm^2^) and a higher area fraction (1.82% *vs.* 1.25%), while maintaining a more refined particle size (410 nm *vs.* 480 nm). These quantitative findings suggest that high-temperature austenitization (925 °C to 1000 °C) facilitates a more complete dissolution of W into the matrix, resulting in a higher degree of supersaturation during subsequent creep [2,10,17]. This increased chemical driving force promotes the prolific nucleation of the Laves phase. The resulting microstructure, characterized by a high density of finely dispersed Laves phase particles, likely provides a superior pinning effect on grain and lath boundaries [1,2].

## 4. Discussion

**Figure 7:**
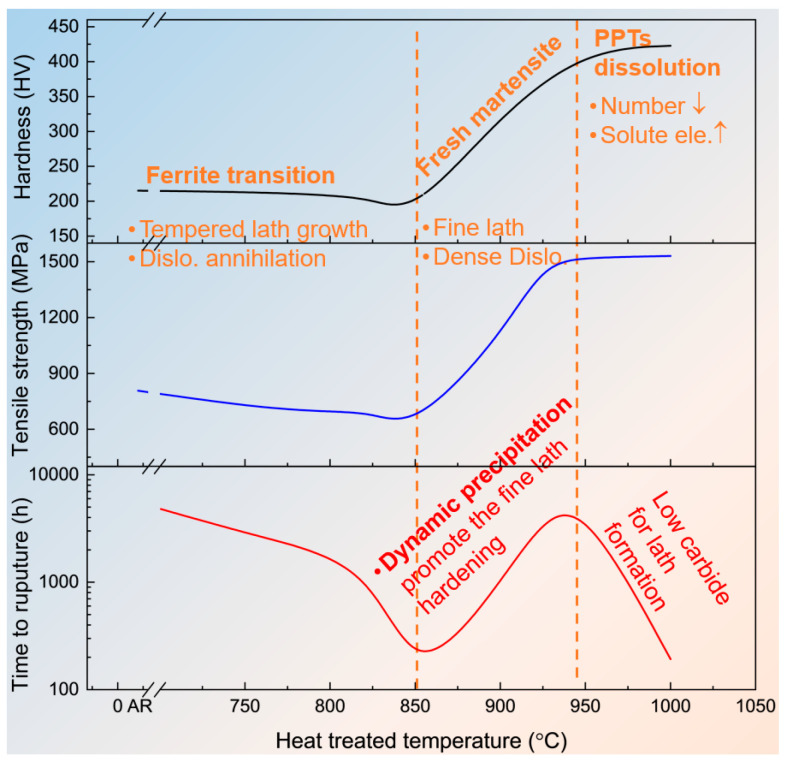
Schematic illustration of the correlation between heat treatment temperature, microstructural evolution, and mechanical properties in T92 steel. The diagram highlights the decoupling between room temperature strength (hardness and tensile strength) and long-term creep resistance. At 850 °C, the matrix undergoes severe recovery into ferrite, leading to minimum strength and creep life. At 1000 °C, the near complete dissolution of carbides yields peak initial strength but results in premature creep failure due to the lack of boundary pinning. In contrast, the 950 °C condition represents an optimal balance, where the synergism between a fresh martensitic matrix, retained M_23_C_6_ anchors, and dynamic Laves phase precipitation provides superior pinning effect to stabilize lath boundaries, thereby extending the creep rupture life.

The evolution of mechanical properties in T92 steel is intrinsically governed by the competition between matrix recovery and phase transformation, as reflected in profiles for both evolutions shown in Figure 7. In the sub-critical temperature regime (760–850 °C), the sharp decline in hardness corresponds to the accelerated tempering of the martensitic matrix. As the temperature approaches the *A_c_*_1_ point, the initial lath structure undergoes severe recovery, transforming into ferrite grains with significantly reduced dislocation density. This microstructural degradation minimizes the Hall-Petch (boundary) strengthening contribution [37], resulting in the minimum hardness observed at 850 °C [10].

Conversely, austenitization above *A_c_*_3_ (900 °C to 1000 °C) resets the microstructure. Air cooling facilitates the formation of fresh lath martensite, where the room temperature strength is primarily dictated by the high density of lath boundaries and PAGBs. These interfaces serve as potent barriers to dislocation motion. Higher temperatures promote the dissolution of carbides, which increases the carbon supersaturation in the matrix and leads to a more refined lath structure upon cooling. These factors, combined with increased solid-solution strengthening, drive the hardness and tensile strength to their peak at 950 °C [26]. Further increasing the temperature to 1000 °C yields no significant gain in strength, as the strengthening effect from dislocation density and boundary refinement reaches a saturation point, as illustrated in the mechanism diagram in Figure 7.

A critical decoupling exists between room temperature strength and high temperature creep resistance. Although the 1000 °C sample possesses the highest initial hardness, its rupture life (192 h) is significantly shorter than that of the 950 °C sample (5422 h). This discrepancy confirms that long-term creep resistance depends on microstructural stability rather than initial hardness, a phenomenon previously noted in studies of Grade 91 and 92 steels subjected to thermal excursions [15].

The superior performance at 950 °C is rooted in a synergistic pinning effect between the fresh martensitic matrix and dynamic precipitation (Figure 7). At this temperature, the remaining stable M_23_C_6_ carbides act as initial anchors for the lath boundaries. During creep, these boundaries provide high density nucleation sites for the prolific dynamic precipitation of fine Laves phase particles and secondary M_23_C_6_. This mutual stabilization maintains the integrity of the lath structure under stress [1]. As shown by the quantitative data in Figure 6H, the high number density of these precipitates exerts a powerful pinning pressure that retards boundary migration and delays the onset of tertiary creep [36].

In contrast, the premature failure at 1000 °C highlights the risks of excessive carbide dissolution. The complete disappearance of stable carbides leaves the boundaries unprotected during the early stages of creep, allowing for rapid matrix recovery before sufficient dynamic precipitation can occur. These findings suggest a strategic trade-off for long term service: the consumption of solute tungsten to form high-density Laves phase precipitates is more beneficial than retaining it in solid solution for short-term strength [17].

Furthermore, the significant scatter in the creep rupture life of 1000 °C samples (192 h *vs.* 1432 h) underscores the stochastic nature of dynamic precipitation in this condition. While the representative 192 h life (validated by the 186 h result at 100 MPa, Figure 2) represents a precipitation lag, catastrophic recovery occurs before effective pinning is established. Conversely, the occasional 1432 h result demonstrates that if intensive precipitation is triggered timely enough to intercept recovery, it can indeed stabilize the fine lath-structure (Figure S1). However, in practice, this extreme inconsistency renders the 1000 °C state unreliable.

From an engineering perspective, the narrow optimal window at 950 °C demonstrates the extreme sensitivity of creep life to austenitization temperature. This underscores the necessity of precise temperature control during PWHT, as a deviation of only 50 °C can drastically reduce creep rupture life despite maintaining high hardness levels. Notably, given the stochastic nature of the 1000 °C specimens, the shorter life (~190 h) must be prioritized for safety-critical design, as the occasional 1432 h result represents an unreliable reinforcement potential. Furthermore, while the 950 °C specimen demonstrates exceptional stability within the current testing timeframe, the well-known slope change in the stress-rupture curves of 9%Cr steels around 10,000 h necessitates caution [38] when extrapolating these findings to long-term service. This relationship between heat treatment, microstructure, and the resulting mechanical performance is summarized in the comprehensive model shown in Figure 7.

## 5. Conclusions

In this study, the effect of heat treatment temperature on the microstructure and creep properties of T92 steel was systematically investigated. The key findings are summarized as follows:

- In the subcritical range below 850 °C, strength decreases as the lath structure evolves into equiaxed ferrite. Upon austenitization between 900 °C and 1000 °C, the formation of fresh martensite drives the hardness and tensile strength back to a peak plateau.
- A fundamental decoupling exists between initial hardness and long-term creep life. The observation that the hardest condition (1000 °C) fails prematurely proves that creep life is governed by the microstructural stability (including dynamic precipitation and lath stability) rather than initial strength. Consequently, initial hardness is not a reliable indicator for predicting the long-term service performance of austenitized T92 steel.
- The superior creep resistance at 950 °C originates from a synergistic stabilization between the martensitic lath boundaries and dynamic precipitates. This optimal temperature provides a martensitic template that facilitates the prolific dynamic precipitation of the Laves phase and M_23_C_6_ carbides, which effectively retard lath boundary migration and extend the creep life.
- The dissolution of carbides during austenitization at 1000 °C leads to premature failure due to the loss of initial boundary protection. Without the pinning effect of pre-existing precipitates, the matrix undergoes rapid recovery before a functional density of dynamic M_23_C_6_ and Laves phase can form to stabilize the structure.
- From an engineering perspective, the extreme sensitivity of creep life to a narrow 50 °C temperature window underscores the critical necessity for precise temperature control during PWHT. Life assessment of power plant components must strictly account for the risks of localized re-austenitization to prevent catastrophic premature failure.

## Data Availability

The original contributions presented in this study are included in the article/Supplementary Materials. Further inquiries can be directed to the corresponding author.

## Supplementary Materials

The following supporting information can be downloaded at: https://www.mdpi.com/article/10.3390/ma19061101/s1, Figure S1: Microstructural characterization of the 1000 °C heat-treated T92 specimen exhibiting an extended creep life (1432 h) at 600 °C/130 MPa. (A) EBSD inverse pole figure (IPF) map showing the effectively preserved martensitic lath structure compared to premature failure samples; (B) BSE image highlighting a higher local density of W-rich Laves phase dynamic precipitation along boundaries; (C) TEM micrograph revealing fine M_23_C_6_ and Laves phase particles pinning the dislocation networks to form the fine lath structure. The localized stabilization is attributed to the stochastic nature of dynamic precipitation during creep exposure, where timely nucleation intercepted the rapid recovery of the fresh martensitic matrix.

## Abbreviations

The following abbreviations are used in this manuscript:

EBSD	Electron backscatter diffraction
TEM	Transmission electron microscopy
BSE	Back-scattered electron
PWHT	Post-weld heat treatment
