# Supplementary material for: Microstructural Evolution Governing the Creep Resistance of Grade 92 Steel Under Wide-Temperature Heat Treatment: From Ferrite Recovery to Dynamic Precipitation Strengthening
Source: Materials (Basel). 2026 Mar 12;19(6):1101. doi: 10.3390/ma19061101 (PMC13028100; doi:10.3390/ma19061101)
Supplement: Supplementary file 1 [file materials-19-01101-s001.zip › materials-4171358-supplementary.pdf]

Supplementary Files

Microstructural evolution governing the creep resistance of Grade 92 steel under wide-temperature heat treatment: from ferrite recovery to dynamic precipitation strengthening

Yinsheng He[1,*], Hongyu Zhou[1], Liming Xu[2] and Keesam Shin[3]

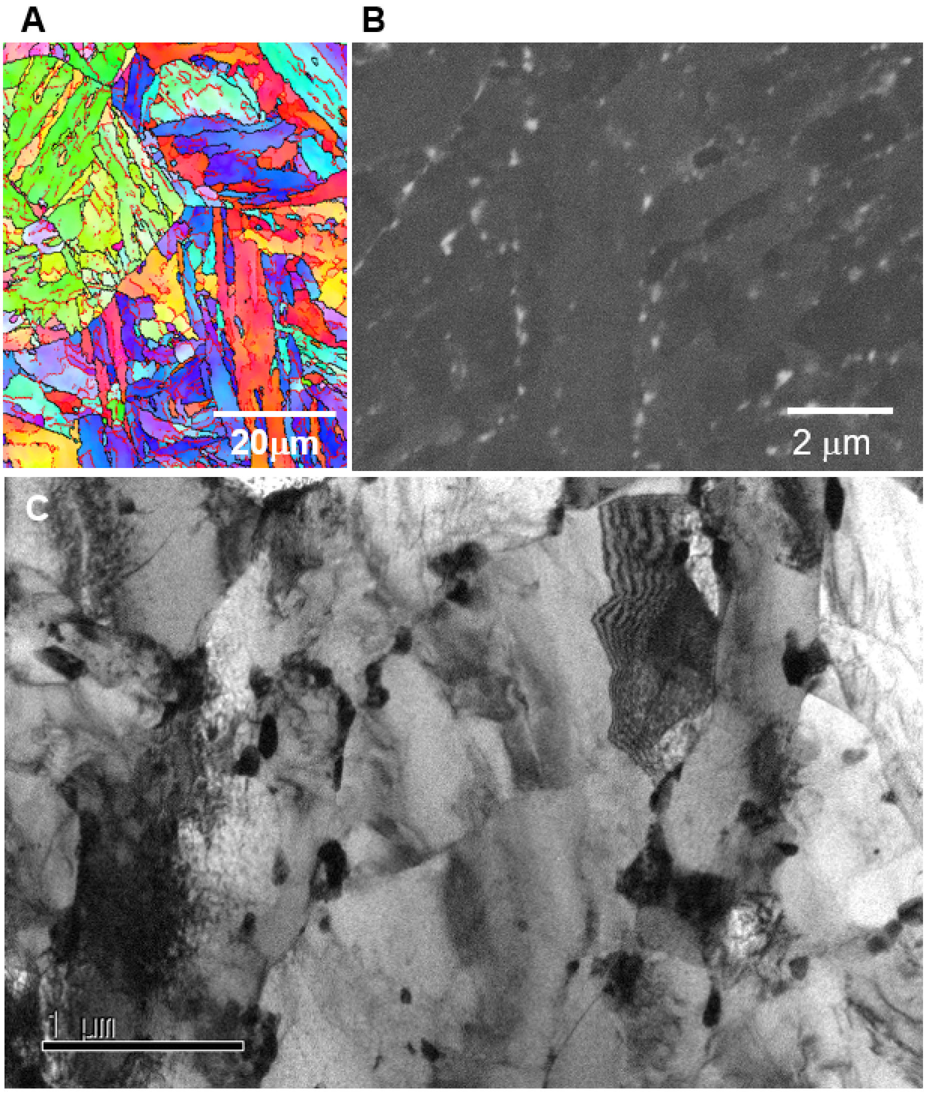

Figure S1. Microstructural characterization of the 1000°C heat-treated T92 specimen exhibiting an extended creep life (1,432h) at 600°C/130MPa. **(A)** EBSD inverse pole figure (IPF) map showing the effectively preserved martensitic lath structure compared to premature failure samples; **(B)** BSE image highlighting a higher local density of W-rich Laves phase dynamic precipitation along boundaries; **(C)** TEM micrograph revealing fine $M_{23}C_6$ and Laves phase particles pinning the dislocation networks to form the fine lath structure. The localized stabilization is attributed to the stochastic nature of dynamic precipitation during creep exposure, where timely nucleation intercepted the rapid recovery of the fresh martensitic matrix.
